# Written action plans for children with long‐term conditions: A systematic review and synthesis of qualitative data

**DOI:** 10.1111/hex.12643

**Published:** 2017-12-09

**Authors:** Andrea Waldecker, Alice Malpass, Anna King, Matthew J. Ridd

**Affiliations:** ^1^ Population Health Sciences Bristol Medical School University of Bristol Bristol UK

**Keywords:** long‐term conditions, paediatrics, qualitative synthesis, self‐management, systematic review, written action plans

## Abstract

**Background:**

Long‐term conditions (LTCs) in children require a high level of self‐management. Written action plans (WAPs) have been advocated to guide decision‐making and support self‐management but there is uncertainty about how WAPs “work” and what aspects are important for successful implementation.

**Objective:**

To review and synthesize existing qualitative evidence about the design and use of WAPs across childhood LTCs.

**Method:**

We undertook a systematic search of the literature (Medline, EMBASE, CiNAHL, PsycInfo, Web of science) from inception to May 2015; critically appraised included studies; and synthesized the findings, drawing on normalisation process theory.

**Results:**

3473 titles were screened and 53 papers read in full. Nine studies (four key, two minor and three of poor quality) contributed to our analysis, predominantly work on asthma from the USA and in specialist settings. WAPs may help to alleviate user worry and boost confidence. Confidence to act was closely linked to feelings of responsibility and authority. The value and use of WAPs are determined by multiple factors, and varies between different user groups. Logistical challenges include sharing a WAP between different stakeholders and keeping it up to date. Colour coding and pictures may enhance the appeal and usability of WAPS.

**Conclusion:**

WAPs are complex interventions but our understanding of their use and value in children with LTCs is limited. WAPs need to meet the needs of users who have different requirements/levels of understanding and confidence according to their different roles. Future research into WAPs needs to be both disease and context‐specific.

## 1 INTRODUCTION

Many long‐term conditions (LTCs) in children, such as eczema, asthma, diabetes and epilepsy, require a high level of self‐management by parents/carers and older children. Fluctuations in disease severity mean often complex treatment regimens must be adjusted daily, without reference to health‐care professionals (HCPs).^1^, ^2^, ^3^, ^4^ It is therefore unsurprising that non‐adherence is the most common reason for treatment failure in the paediatric population.^5^

Written action plans (WAPs) have been advocated to guide parent/carer (hereafter referred to as “carers”) decision‐making and support self‐management. They are especially promoted in school settings to improve the safety of children at risk of emergencies, such as a seizure or hypoglycaemia.^4^, ^6^, ^7^ WAPs exist in many formats but essentially refer to a set of written instructions, which are individualized to the patient and state how to recognize and respond to changes.^8^ They are usually agreed between the carer and the HCP, and held by the carer. The strongest evidence base supporting the use of WAPs is in asthma.^9^, ^10^ However, we have a limited understanding of how WAPs “work” and what aspects of their use are important for successful implementation. Despite their apparent effectiveness in asthma and recommendation in guidelines, WAPs remain under‐used by carers and under‐promoted by HCPs.^11^, ^12^, ^13^, ^14^

To guide the development and implementation of WAPs in other childhood LTCs, we sought to review and synthesize existing qualitative evidence about the design and use of WAPs in this population, seeking to answer the following questions:

1. What is their value and utility for various stakeholders including children, carers, HCPs and school staff?
2. What aspects of WAPs constitute their “active ingredients”?
3. What facilitates and hinders their implementation?
4. What role could they play in the management of diseases other than asthma?

## 2 METHODS

The study protocol was registered with PROSPERO (CRD42015023818) and we have observed the ENTREQ guidelines^15^ on reporting the synthesis of qualitative research. There were three key stages: systematic search; critical appraisal and synthesis informed by normalisation process theory (NPT).^16^

### 2.1 Systematic search

We aimed to identify published qualitative papers with a focus on the views and opinions of any stakeholder on WAPs in the management of childhood LTCs. MeSH headings as well as text words were used in searches which, in summary, combined the following concepts: “children” AND “long‐term conditions” (including specific diagnoses such as diabetes) AND “written action plans” OR “self‐management” AND “qualitative research.” We searched five databases (Medline, EMBASE, CiNAHL, PsycInfo, Web of science) from inception to May 2015. Google and Google Scholar were also searched more informally. We examined reference lists of all potentially relevant papers and contacted the corresponding authors of all included papers for suggestions of any further relevant publications.

Two reviewers screened all abstracts (AW and MR/AM) and read the selected full text papers (AW and MR). To be included, studies had to be qualitative in data generation, analysis and reporting; and have a focus on individualized action plans in children aged 0‐12 years (although studies in school settings where no specific age or school year range was stated were included). Discrepancies were resolved through discussion and reasons for exclusion were documented.

### 2.2 Critical appraisal

AW and AK independently applied the 32‐item COREQ checklist^17^ to the included papers, with an additional question “Was the use of a WAP the main focus of the paper?” Through discussion, papers were categorized (using a modified version of an approach developed by Dixon‐Woods et al^18^) as: key paper (KP) that is conceptually rich and could make an important contribution to the synthesis); “minor paper” (MP) that is methodologically sound but conceptually less rich and relevant to the synthesis); and poor‐quality paper (PQP) that is methodologically weak but may still add to the synthesis, for example studies using open‐ended written questionnaires rather than in‐depth interviews).

### 2.3 Synthesis

Many methods exist for synthesizing qualitative research^19^ and we chose a thematic approach, sensitized by NPT.^16^ NPT has been developed to improve implementation of complex medical interventions, and comprises four broad categories: coherence or sense‐making; cognitive participation; collective action; reflexive monitoring.

First, guided by the NPT concepts we generated questions that we wanted to “ask” of the data (see Table 1). For example, a question under the NPT heading of “collective action” (the work that users do to make the WAP function), was “How will using a WAP affect the daily lives and routines of families?” Next, AW extracted all data (primary verbatim data and secondary interpretations made by the authors) relating to WAPs from the original papers into a Microsoft^®^ Word^®^ document. We then created a Microsoft^®^ Excel^®^ spread sheet, with studies in rows and NPT headings in the columns. Words, sentences or whole paragraphs were then attributed by study to NPT headings. Subcolumn headings were created to represent individual and more nuanced themes. As NPT focuses on pragmatic actions rather on than opinions and beliefs that may inform behaviour, we added an additional column to allow space for data, which would not fit within the NPT headings.

**Table 1:** Research questions, findings and questions for future research (after normalisation process theory [NPT], Murray et al^16^)

NPT heading and description	How this relates to WAPs	Relevant synthesis findings	Questions for future research
Coherence: the meaning and purpose participants see in the intervention and what sense they make of it	What is the perceived purpose of a WAP in the eyes of parents/carers, HCPs and school staff? What do participants think a WAP is or should be? How is a WAP different to other self‐management aids?	Purpose: Information: what to do in an emergency; interpretation of symptoms; identification of affected students Some doctors do not see an added advantage in a WAP over a comprehensive PIL Authority: school nurses are allowed to administer medication, give feedback and liase with clinic staff	Can you see a difference between a WAP and a PIL or instructions on a prescription? Why would having a WAP be useful or not useful to you?
Cognitive participation: how motivated and committed participants are to use the intervention	Can participants easily see the benefits of WAPs and are consequently motivated to invest time and energy into using a WAP? Do participants think the use of WAPs is a good thing?	Motivation: Parents can be motivated by worry that babysitters, teachers, coaches or themselves do not know what to do WAPs get completed because school or camp make it a requirement	Do you think a WAP is a good idea? Why or why not? If you had a WAP, what would you do with it? Do you think a WAP will have an effect on your patients/child?
Collective action: the work participants do to make the intervention function	What do participants have to do for a WAP to function? Do HCPs, carers etc. understand their roles and how they might interact with others in the care of individual children? What might make this effort harder or easier for them? Is the work they have to put into it good value for the outcome?	There can be confusion over who does what and when? Who is the owner or guardian of a WAP? Keeping a WAP up to date requires planning and logistical effort A piece of paper is not sufficient: Parents, school staff and HCPs point out that WAPs need to be part of more comprehensive education Policy needs to support practice, for example allowing capable children to self‐carry medication	Should completing a WAP be part of a routine eczema review? Who do you think should complete a WAP? What would make completing and reviewing a WAP easier for you? How can school staff, parents, children and others change the WAP? How could secondary care, primary care, schools and nurseries coordinate care?
Reflexive monitoring: participants reflect on or appraise the intervention	Do P/C find a WAP useful, keep using it and make sure it is up to date? Do HCPs see a change in the way they conduct patient reviews? How to participants perceive themselves working together with other participants in the care of children? Once participants are using a WAP what do they make of it?	School nurses describe having had positive experiences with asthma action plans, including saving lives School nurses when they get to know the children better would like to change a WAP and feedback to parents and doctors Deviance/modification: Despite being issued with a WAP, parents might discontinue using one or chose not to follow its instructions	How can school staff, parents, children and others influence the content of a WAP? When is time to stop a WAP? How do we know it is no longer needed?
Other (not fitting in NPT framework)	What format and content should a WAP have?	Language needs to be precise Content needs to be free of ambiguity Pictures are liked. Traffic light system seen as easy to read and familiar	

WAPs, written action plans; HCPs, health‐care professionals; PIL, patient information leaflet.

Once complete, AW used mind‐maps to draw out relationships between themes and to identify overarching themes. This process led to the realization that more meaningful themes cut across the NPT headings. MR and AM reviewed the coding, checking for consistency and completeness. AM, AW and MR met regularly to discuss the analysis and emerging themes. In keeping with our thematic approach, we adopted a realist stance, that is that our findings should represent an external reality that would inform future intervention development in this field.

## 3 RESULTS

### 3.1 Literature search and quality appraisal

**Figure 1:**
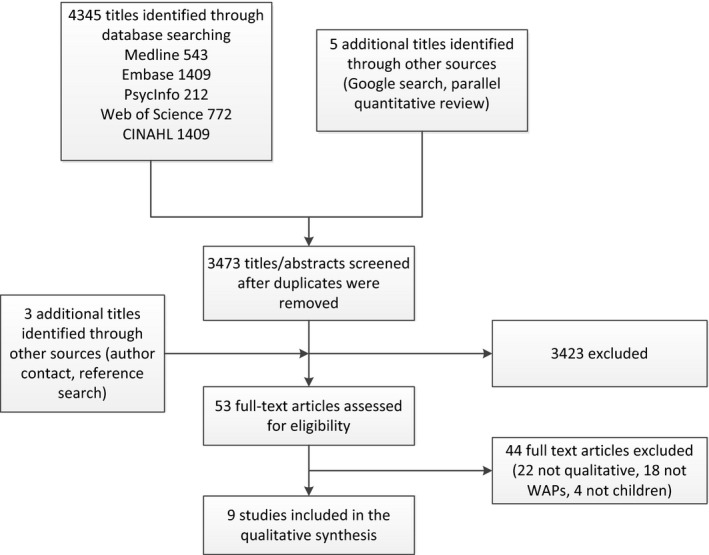
PRISMA flowchart

After removal of duplicate publications, we screened 3473 abstracts and read the full text of 53 papers (see Figure 1). We contacted the authors of four studies comprising children and adults,^20^, ^21^, ^22^, ^23^ where the data were not clearly attributable to children, but none replied so these papers were excluded. Nine papers^24^, ^25^, ^26^, ^27^, ^28^, ^29^, ^30^, ^31^, ^32^ met our inclusion criteria (see Table 2): four papers were judged to be key,^24^, ^27^, ^31^, ^32^ two minor^26^, ^29^ and three of poor quality.^25^, ^28^, ^30^

**Table 2:** Study characteristics

Study First author (year)	Country	No and type of participant	Method of data collection and analysis	Condition studied	Aim
Barton (2005)	Australia	21 caregivers	Single in‐depth interview nested within RCT. Thematic analysis	Asthma	To investigate the attitudes of caregivers towards written asthma action plans
Borgmeyer (2005)	USA	76 school nurses	Questionnaire: open‐ended questions relating to concerns in looking after children with asthma and incidences where an action plan made a difference. “Qualitative items were analysed for themes and patterns …”	Asthma	To explore how school nurses saw their role in caring for students with asthma and how they used asthma action plans
Buu (2014)	USA	10 parents (9 female) 6 shelter staff	One focus group with parents, semi‐structured interviews with shelter staff. Thematic analysis	Asthma	To investigate asthma in homeless children by examining the perspectives of caregivers and shelter staff regarding challenges in caring for children with asthma. One of five domains studied was “asthma action plans”
Egginton (2013)	USA	61, parents 35 school personnel 7 clinic health professionals	Focus groups. Broadly thematic analysis	Asthma	To understand stakeholders' views of the current state of asthma support at school and whether or not asthma action plans might improve current asthma care
Gabeff (2014)	France	479 paediatricians	Questionnaire including open questions. No description of analysis	Eczema	To assess the feasibility and relevance of the personalised written action plan for atopics (developed by Gabeff et al) for paediatricians to use in private practice
Hanson (2013)	USA	65 nurses	Survey with Likert‐type scale and 3 open‐ended qualitative questions. Content theme analysis	Asthma	To assess school nurses' responses to a secure portal designed for the electronic exchange of the asthma action plan between providers and schools and the perceived value of AAPs
Ntuen (2010)	USA	17 paediatricians 8 paediatric dermatologists	Questionnaire with open comments on design attributes and content. “Qualitative” analysis (no further detail given)	Eczema	To assess physicians' perceptions of a written action plan for atopic dermatitis and their openness to using it
Riera (2015)	USA	20 caregivers with limited English proficiency	Focus groups and semi‐structured in‐depth interviews. Constant comparative analysis	Asthma	To develop themes that advance the understanding of the limited English proficiency caregiver experience of caring for a child with asthma and using an asthma action plan and to identify potential areas for future work
Tan (2011)	Singapore	14 parents (13 female)	Focus groups. Thematic analysis using grounded theory	Asthma	To explore the issues pertaining to the parental use of written asthma action plans for their children with asthma

### 3.2 Characteristics of included papers

The characteristics of the nine included studies are summarized in Table 2. Seven studies focussed on asthma and two on eczema (otherwise known as atopic eczema/dermatitis). Five studies used focus groups or in‐depth interviews, the remaining four used open‐ended questionnaires. Collectively, the included papers represent a total of 818 participants, ranging from 14 to 479 per study. Three studies^24^, ^31^, ^32^ represented the views of parents and carers, two school nurses,^25^, ^29^ two studies HCPs,^28^, ^30^ and two studies mixed stakeholder groups of homeless parents and shelter staff^26^ and parents, school nurses, school personnel and clinic health professionals.^27^ Six studies were conducted in the USA, including all of the studies in school settings/incorporating the views of school nurses. There was one study each from Australia, France and Singapore.

### 3.3 Study themes

We identified five themes that cut across the NPT categories: fear and confidence; legitimization and authority to act; valuing and using WAPs; being on the same page—the need for a “liveable” document; and format and content. Table 3 summaries the contribution of each study to these themes, but not the richness of data per theme.^33^ Unless otherwise stated, all quotes refer to stakeholders talking about children with asthma.

**Table 3:** Studies, quality category and theme coverage

Study	Condition	Paper quality/condition	Stakeholder group	Theme				
First author (year)	(A=asthma; E=eczema)	KP (key paper), MP (minor paper), PQP (poor quality paper)		1. Fear and confidence	2. Authority and legitimacy	3. Valuing and using WAPs	4. Being on the same page	5. Form and content
Barton et al (2005)	A	KP	Carers/parents	●		●	●	
Borgmeyer et al (2005)	A	PQP	School nurses	●	●			
Buu et al (2014)	A	MP	Homeless parents Shelter staff	●		●	●	
Eggington et al (2013)	A	KP	Parents					●
			School personnel	●	●	●	●	●
			HCP		●	●	●	●
Gabeff et al (2014)	E	PQP	Paediatricians					●
Hanson et al (2013)	A	MP	School nurses	●	●		●	●
Ntuen et al (2010)	E	PQP	Paediatricians/paediatric dermatologists			●		●
Riera et al (2015)	A	KP	Caregivers/parents with limited English proficiency	●	●			●
Tan et al (2011)	A	KP	Mothers	●		●		

WAPs, written action plans.

#### 3.3.1 Fear and confidence

WAPs can help alleviate parental worry and boost their confidence in looking after their children. Parents across the studies described how having a WAP gave them reassurance and confidence as managers of their child's illness:^24^, ^31^, ^32^

[I find the plan very useful]. *Just to refresh, or you know to know that for sure that we're doing the right thing when you're giving him medication especially when things get worse, or come back to being better* (Parent, p. 145)^24^

A mother with limited English proficiency expressed her increased confidence:

*That plan was among my most important papers (with her birth certificate and passport). Sometimes, I would take it out even though she was not sick and I would look it over to prevent [an exacerbation] from the first symptom she had. It helped me, truthfully. I can honestly say so…* (Mother, p. 21)^31^

Parents also described how having a plan was particularly reassuring and useful when their children were under the care of someone else and liked to give it to schools and extended family.^24^, ^26^, ^31^ Some used the WAP as a tool to motivate the entire family to learn what to do when the primary care giver was not available:^31^

…*We always practiced. Even his little sisters know what to do in the event of an asthma attack…In my house the entire family practices with the child, so that if I'm not there when something happens, everyone knows what to do*. (Mother, p. 22)^31^

Teachers, coaches, school nurses and shelter staff expressed that they would welcome written information on what to do in an emergency involving a child they are looking after.^25^, ^26^, ^27^, ^29^ School nurses knew of specific examples, where the information given in a WAP helped them look after a student and concluded that:

*[the] plan made it easier to determine [the] course of action*. (School nurse, p. 27)^25^

#### 3.3.2 Authority and legitimacy

Confidence to act was closely linked to feelings of responsibility and authority. WAPs may give people the authority to act and to share information about the child. This theme was particularly prominent amongst the studies conducted in school settings,^25^, ^27^, ^29^ possibly because of the responsibility that schools have for their students. Teachers liked that a WAP identified students with asthma and gave credibility to a student's symptoms. This made it easier for a teacher or coach to send the child to the school nurse without feeling pressured to evaluate the child beforehand.^27^

*Teachers need to hear that their responsibility is to believe students and send them [to the nurse]…Often I think teachers do not want to be a pain*. (Teacher, p. 888)^27^

The power of WAPs to give credibility to a child's symptoms was also a view expressed by parents who feared “that teachers did not always “believe” their child was having a problem and just wanted out of class” (p. 888).^27^ Some parents wanted to formalize the school's authority and responsibility, which indicates that they believed that WAPs were a useful way of communicating information about children's medical needs.

*There should be a sign‐off on the AAP [WAP] or a log for staff to report that they have read and understood my child's asthma information*. (Parent, p. 888)^27^

Some studies suggested that the assignment of authority to school nurses and staff can be formalized through a WAP. That is, parental consent for administration of medication, as well as for information sharing, can be a useful component of a WAP.^27^, ^29^

*The informed consent on the AAP [WAP] allowed me to call the Asthma Clinic RN [nurse] to clarify the medication plan*. (School nurse, p. 917)^29^

The WAP also gave school nurses authority to reinforce proper management when a child was not adhering to their treatment plan:^25^

*A student forgot to come to take meds before gym. Exercise induced [an] attack. … showed them the plan where [the] doctor stated two puffs [as] pre‐treatment before gym*. (School nurse, p. 27)^25^

Barton et al^24^ point out that problems may arise where a school or camp requires parents to complete a WAP but parents have not been issued one by their doctor. In this situation, some are “forced” to create a WAP based on their own knowledge and experience, rather than in conjunction with a doctor or nurse.

#### 3.3.3 Valuing and using WAPs

Studies identified several reasons that determined the value and use of the WAP to parents: clarity of instructions or fit to the parents' experience;^32^ parental familiarity with their child's illness;^24^, ^26^ and perceptions of child's self‐reliance.^27^

Tan et al^32^ reported that parents may not stick to a WAP when there is confusion over the interpretation of flu versus asthma symptoms. Consequently, parents may fail to attribute cough as a symptom of asthma and adjust medication accordingly.

*If it is prolonged cough, stretched over 5 days. We have some cough syrup at home. If we give that and it doesn't get better, we bring him to the doctor*. (Parent, p. 186)^32^

The same study also found that parents' threshold to seek medical input varies with their level of confidence and experience both with their child's illness and the WAP.

*…Asthma is simple in the sense that you can give him the ventolin (salbutamol) straight away and you may give him again. If there is continuous coughing, high fever, vomiting, I don't think we can just sit down and follow the plan. We have to rush*. (Parent, p. 186)^32^

In contrast, some parents expressed confidence in managing their child's condition. As parents' familiarity with their child's asthma increased, they no longer felt they needed a WAP.^24^, ^26^ The usefulness of a WAP appears to change throughout a child's disease trajectory and might relate to how reliant the parent feels on it.

*I already know, not that I know everything, but I already know what to do for her, so if something is different or out of our element, I go straight to the doctor…I wouldn't even think to read a piece of paper and see what to do next*. (Parent in homeless shelter, p 145)^26^

Caregivers who possessed written instructions but no longer used them felt that the WAP was not relevant because “*they knew what to do*,” had “*familiarity with the plan*,” or because of “*knowing their child*” (p. 144).^24^ None of the studies representing the views of school staff showed that school nurses and teachers would also become familiar to the extent that they no longer required or wanted WAPs for their students.

Physicians did not always see the additional usefulness of a WAP to information leaflets and so had failed to complete one in the first place. When asked whether they would use an eczema WAP, paediatric dermatologists indicated that they relied on standard patient information leaflets:^30^

*We have our own handout which is extensive and answers other questions such as side effects of steroids, food allergies etc*. (Paediatric dermatologist, p. 31)^30^

In contrast, all general paediatricians in the same study appeared keen to use a WAP for children with eczema.

Instructions may not be followed because they are not understood. Five studies^27^, ^28^, ^29^, ^31^, ^32^ suggested that provision of a WAP alone is not sufficient to enable people to provide appropriate care for a child and that there was a need for training to understand both the WAP and the condition.

*One thing that I like to have with the kids that have asthma: something from the medical provider telling me how much this kid can exercise. Where is the threshold?* (Teacher, p. 888)^27^

*A coach reported that he kept spare inhalers and counted on the students knowing how to use them*. (p 891)^27^

#### 3.3.4 Being on the same page: the need for a liveable document

At nursery, school and during outside activities, children encounter many different people who need to know about their LTC and for whom a WAP might be helpful. Four studies^24^, ^26^, ^27^, ^29^ identified logistical challenges that relate to sharing a WAP between different stakeholders and of keeping it up to date. They reflect a desire “to be on the same page”^29^ without offering practical solutions. While the problem of WAPs becoming “out of date” is acknowledged, reasons for this are not explored:

*He's had this one since October 2001, um, April 2002 (the GP) was going to try and reduce the Seretide but we haven't got that far, so, we're still on this until we can reduce the Seretide, so um. Oh he's on Singulair as well, so, but it's not written on there*. (parent/p. 145)^24^

Three of these four studies focused on schools^27^, ^29^ and homeless shelters.^26^ and sharing a WAP clearly becomes more of a challenge when “third parties” are involved. The studies in school settings show that there is a significant degree of confusion regarding who holds responsibility for a WAP.

*Nurses are not clear what their role is in initiating processes like updating an asthma plan. Physicians were unsure if the AAP* [Asthma Action Plan] *had to be redone every 12 months or redone at the beginning of each school year, even if it had not been 12 months. Parents did not know what all they had to sign to make sure the child could keep medicines at school, carry their own asthma medicines when they were old enough, and allow their child's physician to directly send the AAP to school*. (p. 888)^27^

A concern shared by school and shelter staff was that parents might not pass a copy of a WAP onto them,^26^, ^27^ and “chasing” a WAP can take considerable effort and time:

*Every year you know you will have to call over and over to get the asthma action plan in and all the paperwork filled out*. (School nurse, p. 887)^27^

Egginton et al,^27^ who looked at WAP use in school settings, but included the wider context of parents and HCPs, found that all stakeholders favoured the WAP being passed directly from clinic to school.

School nurses in one US study^27^ said they sometimes become more familiar with a child's asthma than the child's parents or physician and often identify when the WAP no longer matches the child's requirements.

*Can the AAP be a liveable document? Where it's kind of give and take and we can say “this isn't working,” or “something has got to change.”* (School nurse, p 888)^27^

#### 3.3.5 Form and content

Five studies^27^, ^28^, ^29^, ^30^ specifically reported on the desired format and content of WAPs. Two studies on eczema focused on the opinions of paediatricians on form and content.^28^, ^30^

### 3.4 Format

Carers, school staff and HCPs appreciated the “traffic light code” adopted by most asthma WAPs, where green, yellow and red correspond with descriptions of being well, things getting worse and emergencies, respectively, and include instructions of what to do in each category. It was perceived as consistent and clear.^27^, ^29^, ^30^

*Following the different colour codes made it easy for the teacher and EMTs [emergency medical technicians] to navigate. The plan is a consistent way of knowing what to look for and being familiar with the layout*. (School nurse, p. 918)^29^

*…like the use of colors to break up plans based on severity of skin disease*. (Physician, p. 31)^30^

A WAP on a single page,^30^ with the potential to be easily displayed on a fridge,^31^ were also seen as desirable characteristics.

### 3.5 Language

Participants said that the language used in WAPs needed to be simple, specific and easily understood by lay people,^27^, ^30^

*The school personnel universally stated they needed a simple document to provide information of ‘what to do' if a child has an asthma problem*. (p. 887)^27^

*the phrase “moisturize a little extra' is vague”* or *“section on using hydrocortisone needs clarification.”* (Paediatric dermatologists, p. 31)^30^

One study highlighted the importance of the WAP to be language‐concordant, that is written in a language the user understands and speaks. A language‐concordant WAP can then serve as a tool to reduce communication barriers.^31^

*The first asthma plan was given in English. That was the toughest part. Later they got one in Spanish and it was then that things started to get better for her and me…* (Spanish speaking mother, p. 21)^31^

*I would try to read it. I would try to do what it said but I did not understand*. … *My husband does not speak English either, so I did not have anyone to help me. So, my method, my action plan, was to rush to the emergency room.”* (Spanish speaking mother, p. 22)^31^

### 3.6 Pictures

Photographs or drawings are seen as a universal way of communicating.^31^ Especially in eczema, they are appreciated for helping to explain levels of severity.^30^

“*a couple of illustrations of mild vs severe would be helpful”*, “*need some pictures*”, and “*document could use some visuals to break up the text”* (Paediatricians and paediatric dermatologists asked to comment on a sample eczema WAP, p.31)^30^

### 3.7 Content

Desired content in a WAP included: usual medication, rescue medication, triggers and allergens, allowed exercises, how to treat exacerbations, when to call for help and a section to give written consent to school staff.^27^, ^29^ Specialists had a tendency to criticise the content of a WAP if it was different to their usual practice.^30^

One study of an eczema action plan described that the content and format depended on whether the physicians thought the purpose of a WAP to convey information or to aid communication.

*Some saw the WAP [for eczema] as an information tool and demanded that it included further information about topical corticosteroids, hygiene advice, food allergies, instead of the allergy assessment, vaccinations. Others have seen it as a tool for communication or education, desiring less text and more pictures*. (p. 705)^28^

In summary, the “traffic light system” of many asthma action plans was seen as appropriate; the language needs to be kept simple and free of ambiguity; pictures were thought to be especially desirable in eczema; and the content should include, amongst other items, maintenance and emergency treatment.

## 4 DISCUSSION

### 4.1 Summary of findings

We have identified and described five themes that highlight the role that WAPs can play in enhancing the confidence of carers to look after their children and to trust others to look after them. WAPs can give credibility to symptoms and, particularly in school settings, are a means of giving authority to the “owner” to take appropriate action. The most prominent reason for not following the directions on WAPs was that of carers' confidence with their child's illness and its treatment. Problems in their use can also arise if there are language barriers, ambiguity in the instructions or if understanding of the condition itself is limited. Several important aspects regarding the format and content of the WAP are described. Sharing a WAP between different stakeholders and keeping it up to date poses logistical challenges that may hinder the use of WAPs. In the words of a school nurse,^27^ a WAP should ideally be a “liveable” document that all stakeholders can use and amend.

### 4.2 Strengths and weaknesses

This review and synthesis is based on a systematic search to identify relevant qualitative research for all childhood LTCs. Abstracts and full texts were independently screened. Searching for qualitative literature about children with LTCs was challenging, and while it is possible that we failed to include relevant studies, we think the limited number of studies reflects the sparseness of evidence on this topic. A further search (AW, August 2016) for publications since May 2015 did not identify any new studies.

All the authors read the studies, and discussed and agreed the themes, strengthening their validity. Consequent of our broader research questions and our different methodological approach to synthesizing the findings, this paper builds upon a previous relevant review^34^ and presents new insights. NPT provides a robust sociological theory that has been widely promoted as a means to understand implementation, embedding and integration of changes in health care.^16^ It can help analyse implementation processes and inform recommendations to guide implementation work.^35^ We did not, however, allow the NPT framework to constrain our interpretative analysis and the five themes described above cut across the four NPT headings. Overall, we found it a helpful framework, “sensitizing” us to some of issues that emerged (or were missing) in the literature. Furthermore, we have used it to propose specific questions that warrant exploration in future research (see Table 1 and Practice and research below).

Our findings were limited by the conditions and settings in which they were conducted: the literature is dominated by research about asthma (7/9 studies) undertaken in the USA (6/9); and two studies focused exclusively on school nurses, one on caregivers with limited English, and one on homeless children. Given the lack of more general research in this field, this emphasis on minority groups is surprising. Physician's views, especially those of doctors working in primary care, are under‐represented. Some papers (eg Jones et al^36^) focusing on this stakeholder group were excluded because they included adults as well as children.

Lastly, the quality and reporting of methods in the included studies were disappointing: three papers were rated as poor quality; and four papers reported free text responses to self‐administered questionnaires. The quality appraisal process we adopted did not lead to papers being excluded but helped to ensure that the PQPs did not contribute disproportionately to the analysis.

### 4.3 Findings in the context of the literature

In their model of care planning, Burt et al^37^ suggest that care plans may represent an extension of the medical record, a guide to action, and/or as a contract between patients, providers and the health‐care system. They go on to describe three dimensions: perspective (whether the patient or professional is the “target” of the care plan); scope (the degree to which the plan is focussed on discrete behaviours or broader goals); and network (whether care plans are focussed on the core professional‐patient dyad, or a wider care network, for example a multidisciplinary team and/or social networks. WAPs for conditions such as asthma are professional‐centred/behaviour‐focussed, which may be used by people other than the main carers.

While the offer of a WAP may be perceived by patients as an indicator of good care, possession of a WAP does not equate to use.^22^, ^37^ Ring et al,^34^ who undertook a synthesis of the qualitative literature regarding WAP use in asthma, discuss the variable support for and use of action plans, which resonates with our own findings of variation in WAP usefulness, familiarity and reliance.^38^ In further work,^39^ Ring et al^39^ identified multiple barriers reducing the value of WAPs: individual (patient lack of awareness and WAPs not meeting patients' needs; professionals not reinforcing WAP use and/or not involving patients in WAP development) and organizational (many different WAPs in circulation; professionals difficult obtaining paper WAP templates in colour format; knowing whether previously issued WAPs were stored in patients' records or had difficulty accessing these during their consultations; time). Burt et al^37^ also describe the multiple factors serve to mediate (participation and shared decision‐making, supporting self‐management behaviour change and coordinating treatment) and moderate (patient‐level and system‐level issues) care planning. Key practical issues to WAP acceptability and use are apparent in our findings, although our insight into HCPs perspective is limited as is our understanding of the extent to which WAP completion is “shared.”

Ring et al^34^ concluded that patients and physicians view LTCs differently and so attribute different types of usefulness to a WAP. For a WAP to be successfully administered, Ring et al judged that it needed to bridge the gap between patient (asthma as a recurrent acute disease) and physician (asthma as a chronic disease requiring ongoing daily care) perspectives of the condition. By contrast, most parents in our review seemed to value the usefulness of a WAP for day‐to‐day management of chronic over acute symptoms. Further work by Ring et al^39^ found agreement between adults with asthma and HCPs about the suitability of WAPs. Participants thoughts its value depended on time since diagnosis, severity and patient interest/motivation. However, they supported their use by “vulnerable people,” such as the newly diagnosed, children and pregnant women. In short, we agree that WAPs should be targeted: the value of a WAP will vary across different users and at different time points.^40^

If we want to understand the effectiveness of WAPs, their use needs to be studied in the wider context. WAP use may be promoted through improved continuity of care provider and longer appointments.^41^ Even then, primary care practitioners may feel unsure of their roles and responsibilities in supporting children with LTCs.^41^ Caregivers of children with LTCs manage a complex balancing act of competing concerns, which includes weighing up beliefs about the condition against positive or negative beliefs about the treatment and other barriers to treatment.^42^ Parents of children with LTCs present with a range of information needs, communication preferences and motives.^40^, ^41^ Finally, action plans to support self‐care developed in collaboration with health‐care staff are not necessarily then followed by the child's school.^43^ Indeed, schools can create barriers to self‐management because of a lack of understanding and awareness, which may include not allowing children/young people to keep their medication with them.

## 5 CONCLUSIONS

WAPs are complex interventions that require context‐specific development, implementation and evaluation. Their role in helping carers self‐manage children with LTCs remains an incompletely explored concept. WAPs have different value to the numerous stakeholders at different times. They may have a role to play in reconciling differences between clinicians and carers in their view of LTCs as intermittent or ongoing diseases, as these views may influence carer use of treatments and when they seek HCP review. Rather than being viewed as a way of substituting for interaction with health‐care providers, WAPs are one component of several elements that can be brought together as a means of supporting patient self‐management. While individually tailored to a child, the wider process of implementing WAPs needs to be tailored to the context in which they will be used.

### 5.1 Practice and research implications

Here we relate our findings back to the four initial research questions and identify areas in need of further research (Table 3).

What is the value and utility of WAPs for various stakeholders? Parents appear to attach greatest value to a WAP at the beginning of their child's “disease journey.” Later, when parents have internalized the knowledge, WAPs may become superfluous for their own needs but remain useful when the child is in the care of others less familiar with the child and their illness. By contrast, the value of WAPs to HCPs is poorly understood and the views of older children not considered at all.

What aspects of WAPs constitute their “active ingredient”? To our surprise, this issue was not addressed in any of the included studies and research to delineate the different work involved, including influence of the patient‐clinician relationship on its effectiveness, is needed. This should include how the process of completing a WAP with a HCP might influence health‐related behaviour and/or their future use of a WAP; and exploring how the use of a WAP might influence not only the HCPs' own confidence in looking after children with a LTC but also their confidence in the ability of carers to look after the children concerned.

What facilitates and hinders their implementation? At a practical level, sharing of a WAP and the work involved in keeping it up to date was identified as one key challenge, especially when it needs to be shared with multiple parties. Otherwise, data on this issue were thin/absent, partly reflecting the absence of the HCP “voice.”

And what role could they play in the management of diseases other than asthma? We were surprised that WAPs do not appear to be studied in any other LTCs of childhood condition other than eczema. More work may be needed to identify issues specific to different conditions, but possibly there is enough common ground for WAPs for other diseases to be developed based on the findings of this and other reviews.

In practice, patients will probably agree with much of what we present in this paper, but clinicians are likely to be frustrated that their viewpoint is under‐represented and that there is still much uncertainty in this area. Future research needs to build on this existing, if limited, knowledge and explore the views and opinions of all potential stakeholders, within the broader arena in which self‐management is advocated and delivered.

## CONFLICTS OF INTEREST

None to declare.
